# Contribution of HN protein length diversity to Newcastle disease virus virulence, replication and biological activities

**DOI:** 10.1038/srep36890

**Published:** 2016-11-11

**Authors:** Jihui Jin, Jing Zhao, Yingchao Ren, Qi Zhong, Guozhong Zhang

**Affiliations:** 1 Key Laboratory of Animal Epidemiology and Zoonoses, Ministry of Agriculture, College of Veterinary Medicine, China Agricultural University, Beijing 100193, People’s Republic of China

## Abstract

To evaluate the contribution of length diversity in the hemagglutinin-neuraminidase (HN) protein to the pathogenicity, replication and biological characteristics of Newcastle disease virus (NDV), we used reverse genetics to generate a series of recombinant NDVs containing truncated or extended HN proteins based on an infectious clone of genotype VII NDV (SG10 strain). The mean death times and intracerebral pathogenicity indices of these viruses showed that the different length mutations in the HN protein did not alter the virulence of NDV. *In vitro* studies of recombinant NDVs containing truncated or extended HN proteins revealed that the extension of HN protein increased its hemagglutination titer, receptor-binding ability and impaired its neuraminidase activity, fusogenic activity and replication ability. Furthermore, the hemadsorption, neuraminidase and fusogenic promotion activities at the protein level were consistent with those of viral level. Taken together, our results demonstrate that the HN biological activities affected by the C-terminal extension are associated with NDV replication but not the virulence.

Newcastle disease virus (NDV) is the etiological pathogen of Newcastle disease (ND), one of the most important poultry diseases, causing significant economic losses in the commercial poultry industry worldwide, although it also affects many species of birds^1^. The severity of the disease depends upon the viral strain and the host species. Based on their pathogenicity in chickens, NDV strains are classified into three major pathotypes: velogenic, mesogenic, and lentogenic, representing high, moderate, and low virulence respectively^2^. Velogenic NDV strains cause hemorrhagic lesions in the gastrointestinal tract or neurological and respiratory signs, with high mortality rates in chickens of any age, whereas mesogenic strains reduce egg production in laying flocks and cause respiratory disease in young birds. In contrast, lentogenic strains produce only mild respiratory signs in young chickens or no clinical signs. Despite the use of vaccines since the 1950 s, the threat posed by NDV to commercial poultry still exists.

NDV is a member of the genus *Avulavirus* within the family *Paramyxoviridae*^3^. It has a nonsegmented, negative-sense single-stranded RNA genome, which is approximately 15 kb long and encodes six major structural proteins: nucleoprotein (NP), phosphoprotein (P), matrix protein (M), fusion protein (F), hemagglutinin-neuraminidase (HN) and large polymerase protein (L) respectively, in the order 3′-NP-P-M-F-HN-L-5′ 4,5. Two nonstructural proteins, V and W, are also encoded by the NDV P gene and expressed after RNA editing^6^. Like infections with other members of the subfamily *Paramyxovirinae*, NDV infection is initiated by receptor recognition and the binding of the virion to sialylglycoconjugates on the host cell surface, followed by the fusion of the viral lipid envelope with the membrane of the host cell^5^,^7^. This process is accomplished by the interaction of two viral surface glycoproteins, HN and F^8^.

HN is a multifunctional molecule that is responsible for the attachment of the virus to its sialic-acid-containing receptors, and has neuraminidase (NA) activity to hydrolyze the sialic acid molecules from progeny viral particles to prevent viral self-aggregation^7^,^9^. In addition to these activities, HN promotes membrane fusion through its interaction with the F protein, thereby allowing the entry of viral RNA into the cell^10^,^11^. HN is a type II homotetrameric membrane protein, with the N-terminal transmembrane domain followed by an ectodomain, which includes a stalk region and a large C-terminal globular head domain^5^,^12^. Although mutations in the transmembrane and stalk regions of the HN protein can affect the structure and activities of the protein^13^,^14^, the major amino acids residues involved in the sialic-acid-receptor binding and NA activities are located in the globular head^15^,^16^. The stalk mediates the interaction of HN with the homologous F protein, and affects its fusogenic activity^17^,^18^. The globular head is the major functional domain of the HN protein, and contains the two receptor-binding sites, called “site I” and “site II”^19^. Site I is associated with sialic-acid-receptor binding and neuraminidase activity^7^, whereas site II is associated with receptor binding and fusion, but does not influence the neuraminidase activity of the protein^20^,^21^. Because the HN protein mediates fundamental aspects of NDV infection, it is important in viral virulence and tissue tropism^22^,^23^,^24^,^25^.

When the open readings frames (ORFs) of the HN protein in different NDV strains were compared, it was found that the HN proteins vary in length, and at least nine different length variants have been reported to date: 570 amino acids (570aa), 571aa, 572aa, 577aa, 578aa, 580aa, 582aa, 585aa, and 616aa^26^,^27^,^28^. The 616aa HN protein only occurs in avirulent NDV strains. As the precursor HN_0_ protein, the 616aa requires the cleavage of 45 amino acids from the C-terminus to recover the HN activity^12^. The HN protein of 577aa is present in virulent and avirulent strains, whereas the HN protein of 571aa can only be detected in highly virulent strains. Previous study on the basis of an infectious clone of the lentogenic vaccine virus Clone-30 has shown that the pathogenicity of NDV is not influenced by the length variations in the 571aa, 577aa and 616aa HN proteins^29^. But in the context of the virulent strains or other length variants, especially the unnatural length of HN protein, the data are lacking. Furthermore, these length differences arise from changes in the length of the C-terminal global head, the main functional region of HN, with both receptor-binding activity and neuraminidase activity, and thus they may influence the activity of the HN protein, and even the biological characteristics of the virus. However, there have been very few systematic studies of this issue.

NDV strains are classified into two subdivisions, class I and class II. Class II can be further divided into nine genotypes I to IX^30^,^31^. All genotypes of class II, except IV^32^, are still in circulation, while the genotype VII viruses are the predominant NDV strains throughout the world in recent years^33^,^34^. In this study, we chose a representative virulent isolate of NDV genotype VIId circulating in China (SG10) as the model virus upon which to build the recombinant NDVs encoding truncated or extended HN proteins. We used these to evaluate the contribution of the HN protein length to the pathogenicity, replication and biological characteristics of the virus.

## Results

### Recovery of recombinant viruses and identification of various HN proteins lengths

**Figure 1:**
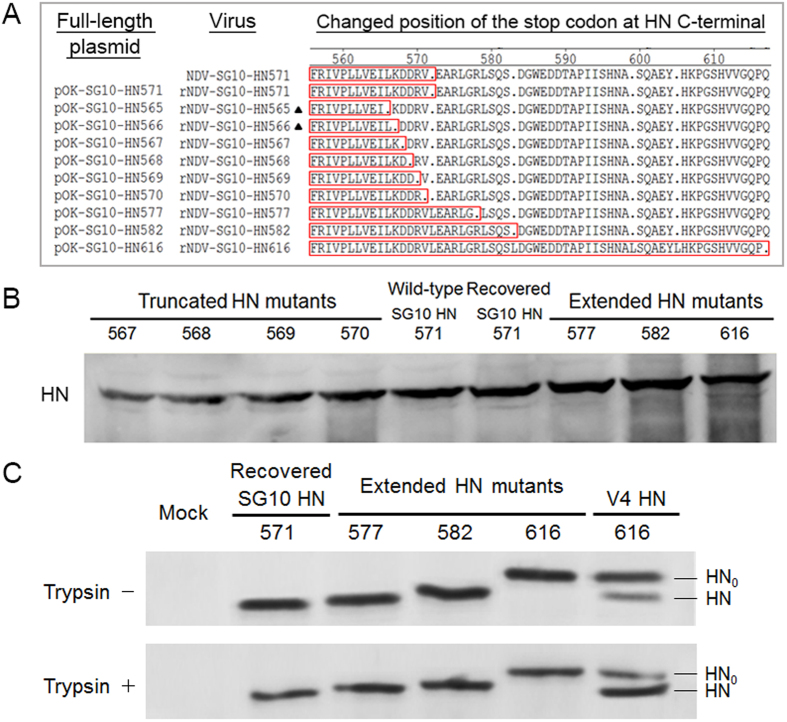
Recovery and identification of recombinant viruses. (**A**) Alteration of the length of the HN C-terminus by site-directed mutagenesis. “·”, stop codon; and amino acids at the carboxy terminus of the HN protein are boxed. The two recombinant viruses that could not be successfully recovered are indicated with black triangles. (**B**) Western blot analysis for HN proteins of recombinant viruses using polyclonal antibody raised against the NDV SG10 strain. (**C**) The effect of protease treatment on the extended HN mutants. Western blot analysis of HN proteins in Vero cells infected with extended HN mutants processed in the presence and absence of trypsin. Mock infection served as negative control and the V4 strain was used as positive control.

To investigate the role of the length of HN protein in NDV virulence and pathogenesis, the full-length plasmids encoding 10 different length variants of the HN protein were constructed to rescue recombinant viruses (Fig. 1A). Nine viable viruses were recovered with these 10 full-length cDNAs and the full-length cDNA encoding the 565aa HN protein did not recover a viable virus. The supernatants from transfected BSR T7/5 cells were passaged twice in embryonated specific-pathogen-free (SPF) chicken eggs. The allantoic fluids with positive HA titers were processed to isolate the viral RNAs, which were subjected to RT-PCR and sequence analyses. Nucleotide sequencing confirmed the presence of the mutations introduced into the HN genes. These results indicated that nine recombinant viruses were rescued, but rNDV-SG10-HN565 was not (Fig. 1A).

To further determine the stability of the rescued viruses, eight rescued viruses excluding rNDV-SG10-HN566, were successively passaged 10 times through 9-day-old SPF eggs, and the mutated regions in the HN gene were sequenced from the allantoic fluid of each passage. However, a sequence revertant to the original amino acid residue was detected in rNDV-SG10-HN566 at the third passage. The sequencing results indicated that the eight rescued viruses were stable, except rNDV-SG10-HN566. Therefore, these eight rescued viruses, together with the wild-type virus (NDV-SG10-HN571), were used in subsequent experiments.

The various lengths of HN protein were analyzed by western blotting using a polyclonal anti-SG10 strain serum. This confirmed that the correct mutant HN proteins were present where expected (Fig. 1B). Furthermore, to analyse whether the extended HN proteins require the cleavage of amino acids from the C-terminus, western blot analysis of extended HN mutants processed in the presence or absence of trypsin was performed. The results showed that these extended HN proteins could not be proteolytically processed in the presence of trypsin (5 μg/ml) in Vero cells. However, the avirulent strain V4 with HN protein of 616aa, could be observed two band (HN_0_ and HN) in the absence or presence of trypsin (Fig. 1C). This demonstrated that protease treatment could not trim off the HN616 protein extension mutations of SG10 strain.

### Pathogenicity of the recombinant viruses

To evaluate the influence of the length of the HN protein on NDV pathogenicity, the wild-type and rescued viruses were examined with the standard pathogenicity tests used for NDV: the mean death time (MDT) test in 9-day-old embryonated SPF chicken eggs and the intracerebral pathogenicity index (ICPI) test in 1-day-old SPF chicks *in vivo*. As summarized in Table 1, the MDT values for all eight rescued viruses ranged from 39.0 to 43.5 h, which were very similar to the wild-type NDV-SG10-HN571 value (42 h), and the results of the ICPI test were consistent with the results of the MDT. The ICPI values for all eight rescued viruses and NDV-SG10-HN571 were >1.90. Based on both the MDT and ICPI values, all these viruses were classified as velogenic strains. When the viruses were used to inoculate BSR T7/5 cells at a multiplicity of infection (MOI) of 0.01 PFU/cell. The cytopathogenic effects (CPEs) were observed in the cells infected with each virus at 48 h post-infection (hpi) (see Supplementary Fig. S1). These results indicate that the length of the HN protein has no obvious effect on NDV pathogenicity.

### Growth properties of the mutant viruses

**Figure 2:**
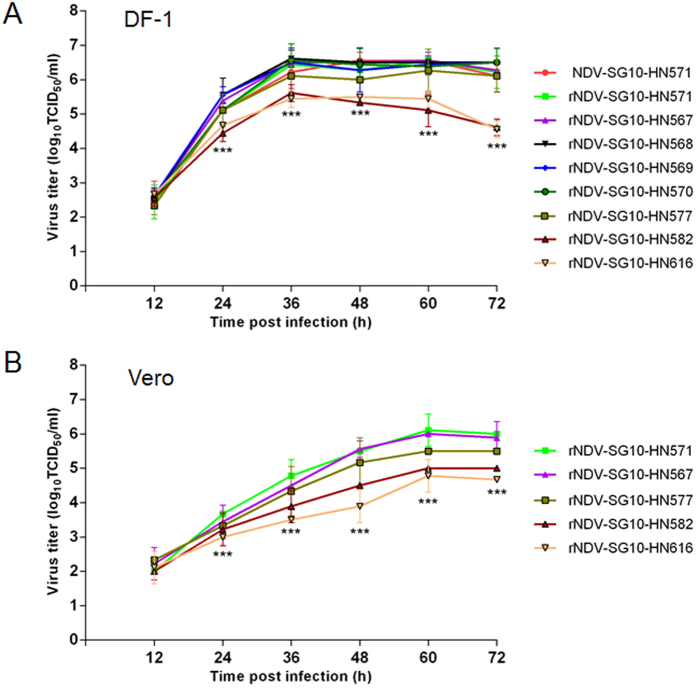
Growth characteristics of recombinant NDVs. Multiple-cycle growth kinetics were used to assess the differences in the growth of these viruses. (**A**) DF-1 cells were infected with rNDV-SG10-HN571, rNDV-SG10-HN567, rNDV-SG10-HN568, rNDV-SG10-HN569, rNDV-SG10-HN570, rNDV-SG10-HN577, rNDV-SG10-HN582, or rNDV-SG10-HN616 at an MOI of 0.01 PFU/cell, and assayed as described in the Methods. (**B**) Vero cells were infected with rNDV-SG10-HN571, rNDV-SG10-HN567, rNDV-SG10-HN577, rNDV-SG10-HN582, or rNDV-SG10-HN616 at an MOI of 0.01 PFU/cell, and assayed as described in the Methods. Briefly, supernatant samples were collected at 12-h intervals for 72 h, and the viral titers were determined with a TCID_50_ limiting dilution assay, and calculated with the method of Reed and Muench. Means and standard deviations were shown from three independent experiments. Asterisks indicate the significance of the difference between the recombinant viral titer and the parental viral titer. P values were calculated with Tukey’s test (95% confidence levels). ***p < 0.001, extremely significant.

To investigate whether the length of the NDV HN protein affects viral replication in cell culture, the growth characteristics of the wild-type and recombinant viruses were evaluated from their multicycle growth kinetics in a chicken embryo fibroblast cell line (DF-1), and an African green monkey kidney cell line (Vero). The multistep growth curves for the recombinant HN mutant viruses in DF-1 cells (Fig. 2A) showed that the replication kinetics of all the HN-truncated mutant viruses (rNDV-SG10-HN567, rNDV-SG10-HN568, rNDV-SG10-HN569, and rNDV-SG10-HN570) were similar to those of wild-type NDV-SG10-HN571 and rNDV-SG10-HN571, whereas the two HN-extended mutant viruses (rNDV-SG10-HN582 and rNDV-SG10-HN616) showed delayed growth and significantly lower viral yields than the wild-type and parental recombinant viruses at 24 hpi (p < 0.001). rNDV-SG10-HN577 replicated to titers that were intermediate between the extremes of rNDV-SG10-HN570 and rNDV-SG10-HN616. To verify the growth characteristics of the recombinant viruses, Vero cells were infected with five representatives of the HN length mutant viruses (rNDV-SG10-HN571, rNDV-SG10-HN567, rNDV-SG10-HN577, rNDV-SG10-HN582 and rNDV-SG10-HN616). As shown in Fig. 2B, the trends in the viral growth curves in Vero cells were similar to those in DF-1 cells, although the growth rates of the five HN mutant viruses in Vero cells were lower than those in DF-1 cells. These results indicate that the HN protein truncation mutations negligibly influenced the replication efficiency of NDV in cells, but that the extension mutations substantially reduced the efficiency of NDV replication.

### Hemagglutination (HA) titers of recombinant viruses

To evaluate the influence of the length of the HN protein on NDV HA titer, HA assay of the five representative HN length mutant viruses were performed. As shown in Table 2, at the virus titers of 7.0 and 6.0 log_10_ TCID_50_/ml, the rNDV-SG10-HN567 HA titer was slightly lower than parental virus rNDV-SG10-HN571, whereas the HA titers of HN-extended mutant viruses (rNDV-SG10-HN577, rNDV-SG10-HN582 and rNDV-SG10-HN616) were higher than the parental virus, especially the rNDV-SG10-HN582 HA titer existed significant differences (p < 0.05). The result suggests that the extension of HN protein leads to a moderate increase for the HA titer of NDV.

### Hemadsorption and neuraminidase activities of the recombinant viruses and expressed HN protein mutants

**Figure 3:**
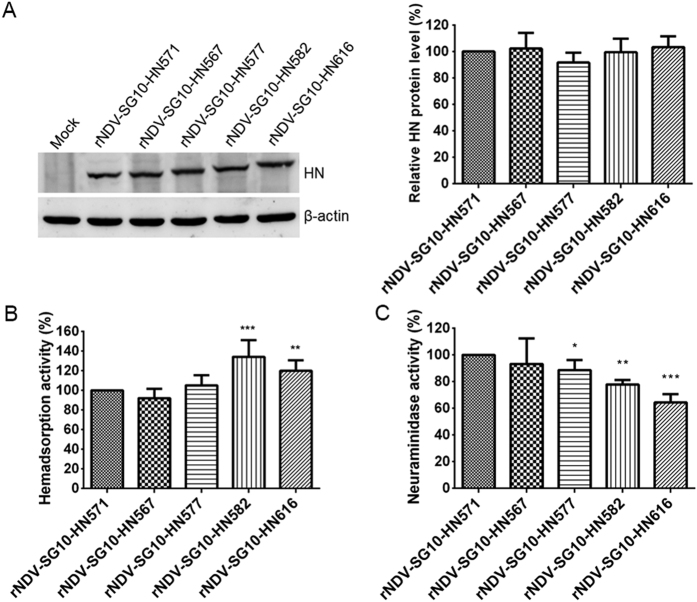
HAd and NA activities of parental and recombinant viruses. (**A**) The amount of each HN protein was determined by western blot analysis at 24 h post-infection of Vero cells by using anti-SG10 serum. HAd (**B**) and NA activities (**C**) were examined in Vero cells infected with the virus at an MOI of 0.1 PFU/cell. HN protein expression level, HAd and NA activities of these viruses are expressed as percentages of the values for rNDV-SG10-HN571, which are deemed to be 100%. Each bar represents the mean and standard deviation of three independent experiments. Asterisks indicate the significance of the differences between the biological activity of a recombinant virus and that of the parental virus. P values were calculated with Tukey’s test (95% confidence levels). *p < 0.05, significant; **p < 0.01, very significant; ***p < 0.001, extremely significant.

**Figure 4:**
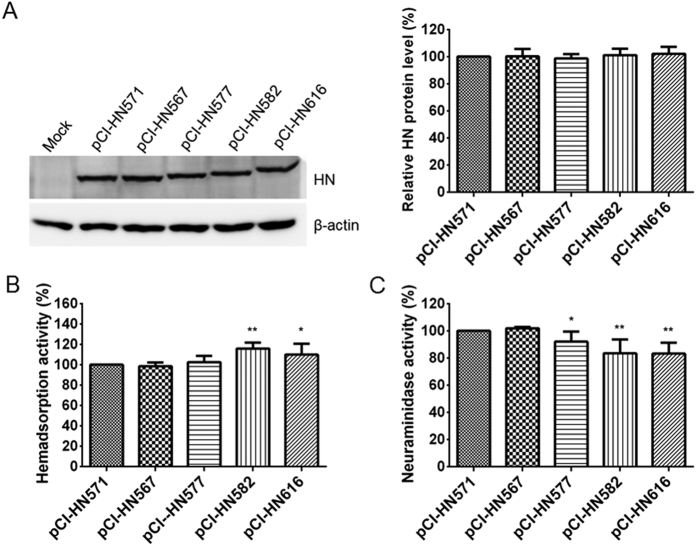
HAd and NA activities of expressed HN protein mutants. (**A**) The amount of each HN protein was determined by western blot analysis at 24 h post-transfection of Vero cells by using anti-SG10 serum. HAd (**B**) and NA activities (**C**) were examined in Vero cells transfected with the 0.5 μg each of pCI-HN mutant plasmids. HN protein expression level, HAd and NA activities of these HN mutants are expressed as percentages of the values for expressed HN571 protein (pCI-HN571), which are deemed to be 100%. Each bar represents the mean and standard deviation of three independent experiments. Asterisks indicate the significance of the differences between the biological activity of an HN protein mutant and that of the HN571 protein. P values were calculated with Tukey’s test (95% confidence levels). *p < 0.05, significant; **p < 0.01, very significant.

To determine whether the length of the NDV HN protein modulates the biological activities of NDV in cultured cells, the five representative HN length mutant viruses were assessed for their hemadsorption (HAd) and neuraminidase (NA) activities. Firstly, the HN protein expression of five representative HN length mutant viruses was measured in Vero cells. The HN expression of the various HN length mutants was similar to that of parental HN (Fig. 3A). Subsequently, the biological activities of each recombinant virus were analyzed and calculated as a percentage of that of rNDV-SG10-HN571, whose biological activities were deemed to be 100%. The HAd activity of rNDV-SG10-HN582 was 134% of that of rNDV-SG10-HN571, which was the greatest increase in all the mutants, followed by 120% for rNDV-SG10-HN616. The HAd activities of rNDV-SG10-HN567 and rNDV-SG10-HN577 did not differ significantly from those of rNDV-SG10-HN571 (Fig. 3B). All the extended HN length mutant viruses showed significantly reduced NA activity relative to that of the parental recombinant virus, with the following values (relative to rNDV-SG10-HN571, 100%): rNDV-SG10-HN577, 89%; rNDV-SG10-HN582, 78%; and rNDV-SG10-HN616, 64% (Fig. 3C). Furthermore, the HAd and NA activities of HN mutants at the protein level were performed using HN expression plasmids (pCI-HN571, pCI-HN567, pCI-HN577, pCI-HN582, pCI-HN616). The result was almost the same as the viral level. The HAd activities of HN582 and HN616 proteins were markedly increased than those of HN571 under the similar HN expression (Fig. 4A,B). The NA activities of all extended HN protein mutants were significantly decreased than those of HN571 (p < 0.05 or 0.01) (Fig. 4C). These results suggested that the extension of HN protein increased the hemadsorption capacity, but reduced the NA activity at viral and protein levels.

### Syncytium formation induced by recombinant viruses and expressed HN protein mutants

**Figure 5:**
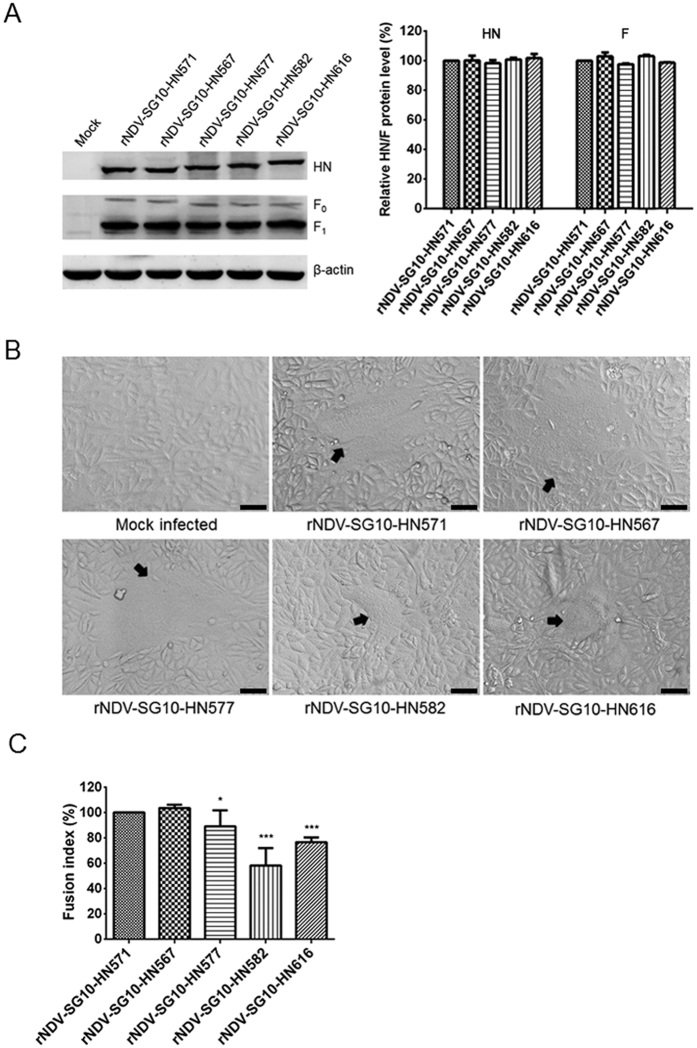
Syncytium formation of parental or recombinant viruses. (**A**) The amount of each HN or F (F_0_ and F_1_) protein was determined by western blot analysis at 48 h post-infection of Vero cells by using anti-SG10 serum or anti-NDV F rabbit polyclonal antiserum. (**B**) At 48 h post-infection, Vero cell monolayers that had been infected with parental or recombinant NDV at an MOI of 0.1 PFU/cell in six-well plates were digitally photographed under an inverted microscope (IX73; Olympus) at ×100 magnification. Black arrows indicate syncytia. Bar = 50 μm. (**C**) The fusion index values of these viruses were calculated as the ratio of the total number of nuclei to the number of cells in which the nuclei were observed. All values are expressed relative to the value for rNDV-SG10-HN571 (100%). Each bar represents the mean and standard deviation of three independent experiments. Asterisks indicate the statistically significant differences. P values were calculated with Tukey’s test (95% confidence levels). *p < 0.05, significant; ***p < 0.001, extremely significant.

**Figure 6:**
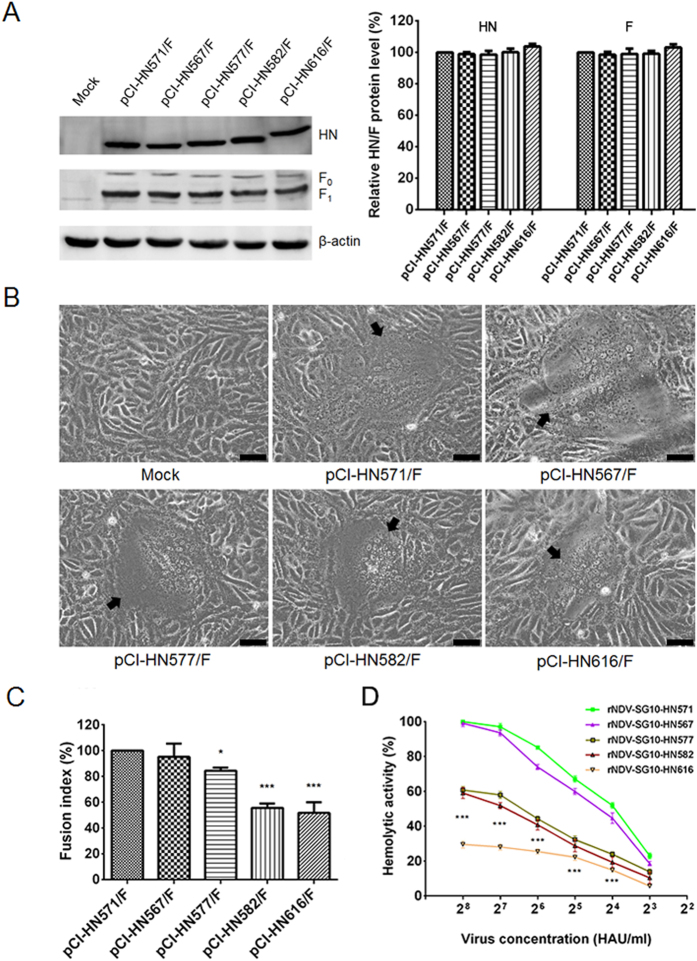
Syncytium formation in Vero cells co-transfected with expressed plasmids and hemolytic activities of parental or recombinant viruses. (**A**) The amount of each HN or F (F_0_ and F_1_) protein was determined by western blot analysis at 48 h post-transfection of Vero cells by using anti-SG10 serum or anti-NDV F rabbit polyclonal antiserum. (**B**) At 48 h post-transfection, Vero cell monolayers that had been co-transfected with 1 μg each of pCI-F and pCI-HN mutant plasmids in six-well plates were digitally photographed under an inverted microscope (IX73; Olympus) at ×100 magnification. Black arrows indicate syncytia. Bar = 50 μm. (**C**) The fusion index values of these HN mutants were calculated as the ratio of the total number of nuclei to the number of cells in which the nuclei were observed. All values are expressed relative to the value for pCI-HN571/F (100%). (**D**) The hemolytic values for all the viruses were expressed as percentages of the values for rNDV-SG10-HN571 at 2^8^ hemagglutination unit/ml, which were considered to be 100%. Each bar represents the mean and standard deviation of three independent experiments. Asterisks indicate the significance of the difference between the fusion index of a recombinant virus and that of the parental virus. P values were calculated with Tukey’s test (95% confidence levels). *p < 0.05, significant; ***p < 0.001, extremely significant.

First, western blot analysis based on protein lysates, derived from Vero cells infected with recombinant viruses, indicated that the HN and F (F_0_ and F_1_) expressions of the various HN length mutants was similar to that of parental virus (Fig. 5A). Under these conditions, a fusion index assay was performed to measure the ability of the parental and recombinant viruses to induce syncytium formation in Vero cells. All the HN extension mutant viruses decreased the fusion ability in Vero cells, and the syncytia induced by rNDV-SG10-HN577, rNDV-SG10-HN582 and rNDV-SG10-HN616 were markedly smaller than those induced by the parental virus (Fig. 5B). Quantification of the fusogenic abilities of the mutants (fusion index compared with the parental level) showed that rNDV-SG10-HN577, rNDV-SG10-HN582, and rNDV-SG10-HN616 had significantly reduced fusogenic activity (89%, 58%, and 77%, respectively) (p < 0.05 or 0.001) (Fig. 5C). Subsequently, the fusogenic promotion activities of truncated or extended HN mutants at the protein level were performed using expression plasmids pCI-HN571, pCI-HN567, pCI-HN577, pCI-HN582, pCI-HN616 and pCI-F. The result was almost consistent with the viral level. The HN577, HN582 and HN616 showed significantly reduced fusogenic promotion activity (84%, 56%, and 52%, respectively) compared with HN571 under the similar HN and F expression (p < 0.05 or 0.001) (Fig. 6A–C). These results indicate that the extension of the HN protein seriously impairs the fusogenic ability at viral and protein levels, whereas truncation mutations of the HN protein have no statistically significant influence on the fusogenic capacity.

### Hemolytic activities of the recombinant viruses

To evaluate the influence of the length of the HN protein on NDV hemolytic activity, five representative HN length mutant viruses were tested at virus concentration ranged from 2^3^–2^8^ hemagglutination unit/ml (HAU/ml). As shown in Fig. 6D, the hemolytic activity of rNDV-SG10-HN567 was slightly lower than parental virus rNDV-SG10-HN571, whereas HN-extended mutant viruses (rNDV-SG10-HN577, rNDV-SG10-HN582 and rNDV-SG10-HN616) were significantly decreased than that of rNDV-SG10-HN571 (p < 0.001). The rNDV-SG10-HN616 hemolytic activity had the greatest reduction in all the mutants. These results indicate that the extension of the HN protein mutations seriously impairs the hemolytic activity, which is consistent with the fusogenic ability at viral and protein levels.

## Discussion

The HN protein of NDV plays important roles in viral invasion and maturation, with three distinct activities: receptor binding, NA activity (receptor cleavage) and fusion promotion^18^,^35^,^36^,^37^,^38^. The HN protein has been shown to contribute greatly to NDV pathogenesis^22^,^23^,^39^. Previous studies have also suggested that even a single point variation in the different regions of the HN protein can markedly affect the biological activities of the protein or the virus^40^,^41^,^42^,^43^,^44^. In addition, for other paramyxovirus, measlesvirus attachment (H) protein stalk-length variation through deletion or insertion of heptad repeat (HR) elements at positions flanking this section including residues 111, 114, and 118 could alter fusion complexes function^45^. Vesicular stomatitisvirus (VSV) is a nonsegmented, negative-sense single-stranded RNA virus, as well as NDV. Changes in the transmembrane domain (TMD) length of VSV spike protein G could affect secretory transport dynamics and the capacity of the transport machinery to concentrate cargo^46^. However, the contribution of HN protein length diversity to NDV has not been thoroughly investigated. In this study, a series of recombinant NDVs containing truncated and extended HN proteins were generated with a reverse genetics technique to evaluate the contribution of the HN protein length to the pathogenicity, replication and biological characteristics of NDV *in vivo* and *in vitro*. The extension of the HN protein increased the HA titer, HAd activity and reduced the NA activity, fusion index, hemolytic activity and replication capacity of NDV, without any observable changes in viral pathogenicity.

The HN protein length diversity of NDV mainly arised from differences in the position of the stop codon of the HN ORF^26^,^27^,^28^. To explore the distribution of various HN protein length in NDVs of different clades and virulence, we analyzed all NDV HN sequences available in the National Center for Biotechnology Information (NCBI). As shown in Supplementary Table S1, there were 12 different HN protein length, with different length showing a clade and virulence preference. Most class І (76.1%) and class II genotype І (75.4%) viruses had HN protein of 616aa, 89.5% of class II genotype II viruses had HN protein of 577aa, and HN protein of class II genotype III (100%), IV (100%), V (100%), VI (97.9%), VII (92.3%), VIII (50.0%), IX (96.2%) contained 571aa. The virulence of 347 isolates is known, 84.8% of these virulent isolates have HN protein of 571aa, while most low-virulence strains contain HN proteins of 577aa (33.0%) and 616aa (56.7%). In the present study, a genotype VIId NDV strain (SG10) with a 571aa HN protein was used as the model virus to construct the recombinant NDVs encoding truncated or extended HN proteins^47^. The MDT and ICPI results indicated that mutations affecting the length of HN did not alter the virulence of the mutant viruses in embryonated chicken eggs or 1-day-old chickens. These results are consistent with a previous report of an avirulent NDV strain (Clone-30) with a 577aa HN protein^29^. This suggests that NDV pathogenicity is not influenced by the length of the HN protein, in either velogenic or lentogenic strains. The 570aa HN protein is the shortest length detected, as previously reported. A recombinant NDV expressing a 567aa HN protein (rNDV-SG10-HN567) was successfully recovered in this study. During the rescue of the recombinant virus, a recombinant NDV containing a 565aa HN protein (rNDV-SG10-HN565) was not recovered and a sequence revertant of rNDV-SG10-HN566 was recovered after passage in embryonated SPF chicken eggs. These results indicate that the length of the HN protein cannot be reduced indefinitely, and we suppose that this may relate to the evolution of NDV.

The Ulster, Queensland, V4 strains or some other avirulent strains with HN616, produce an HN protein precursor (HN_0_ protein) which requires proteolytic activation before the virons can attach to the receptors on host cells^48^,^49^. Schuy *et al*. reported that the HN_0_ protein of the Ulster strain was cleaved by trypsin *in vitro* and *in vivo* to generate the HN protein of approximately 571 amino acids^50^. In the present study, the extended HN protein of rNDV-SG10-HN616 was not cleaved by trypsin *in vitro*. These may well be closely related to the extended amino acid sequences and NDV virulence. The originally protein non-coding region was forced to be a coding region. Hence, the extended amino acid residues are likely to be unnatural. The extended 45 amino acid residues of rNDV-SG10-HN616 and avirulent strains with HN616 vary considerably with homology <45%. In addition, rNDV-SG10-HN616 is still a virulent strain. The results are in accordance with the viewpoint that only some avirulent NDV strains produce an HN_0_ protein precursor^12^,^48^,^49^.

The growth characteristics of the recombinant viruses containing a series of truncated or extended HN proteins were assessed based on their multicycle growth kinetics in DF-1 and Vero cells. The recombinant viruses expressing extended HN proteins (577aa, 582aa, and 616aa) showed reduced viral titers *in vitro* compared with those of the parental virus and the recombinant viruses containing truncated HN proteins. Interestingly, the reduced efficiency of viral replication in the cells did not reduce the virulence of NDV. These data seem to contradict the consistency between NDV replication and pathogenicity previously reported^51^,^52^, but probably attributable to differences in viral reproduction *in vivo* and *in vitro.* For example, Kim *et al*. reported that mutations at the fusion protein cleavage site of avian paramyxovirus conferred increased replication *in vitro* but did not increase its replication or pathogenicity in chickens and ducks^53^. Therefore, future studies should focus on the replication capacity and tissue tropism *in vivo* of recombinant viruses containing HN proteins of different lengths. What is more, these results seem to suggest that the replication ability of these viruses is not closely related to their pathogenicity. And the data are similar to the report that NDV M protein affected viral replication *in vitro*, but only had a minor effect on virulence^54^. Although the exact mechanism is not clear, it is likely to be related to the balance among the HAd, NA and hemolytic activities which ultimately determines the outcome of infection. We suppose that the decrease of NA and hemolytic activities mainly results in the impairment of replication ability, while the increase of HAd activity is likely to maintain the NDV virulence. Another plausible explanation is that when viral replication reaches a certain level, the higher virus titer does not have greater impact on the virulence. In this study, all the viruses maintain at high replication levels although the different virus titers exist. Thus, the replication of the two HN-extended mutant viruses (rNDV-SG10-HN582 and rNDV-SG10-HN616) was decreased almost 1.0 log10 compared with the parental strain at 24 hpi in DF-1 and Vero cells, but it is insufficient to alter the virulence.

Our *in vitro* studies of NDV support the hypothesis that the length of the HN protein affects some biological functions of the virus. Extension of the HN protein increased NDV HAd activity, reduced its NA activity and fusion index. These results emphasize the importance of the 571aa HN protein in the NA and fusogenic activities of NDV strain SG10. The impairment of the NA and fusogenic activities induced by the extension of the HN protein is consistent with the viral replication observed in cells. However, the HAd activity of HN appeared to be independent of its NA and fusogenic activities. Our results suggest that these altered activities could affect viral growth *in vitro* and the extension of HN allows viral attachment but prevents virion release during the life cycle of NDV.

Extension of the HN protein likely reduces the interaction between HN and F, leading to the lower fusion indices and hemolytic activities of the recombinant viruses expressing extended HN proteins. The exact mechanism is unclear, but it is likely to be related to the second receptor-binding site (site II), which is engaged by the C-terminal extension of the NDV HN protein, because it plays an active role in F activation^10^,^19^,^55^. Our data showed that the recombinant viruses containing HN extension mutations displayed substantially reduced viral titers *in vitro*. It is possible that, although these viruses had higher receptor affinity, their fusogenic and NA activities were diminished, reducing the efficiency of their replication in infected monolayers. Interestingly, the extension of the HN protein did not alter NDV virulence, as shown with MDT and ICPI tests, which seems to indicate that the fusogenic and NA activities of NDV are not related to its pathogenicity, or that these various assays have different sensitivities. These results also suggest that the three functions of HN are flexible and balanced at the virus level.

In summary, the contributions of HN protein length to the pathogenicity, replication and biological characteristics of NDV were investigated using recombinant viruses expressing truncated or extended HN proteins, which were rescued with a reverse genetics method. The extension of the HN protein affected the hemadsorption, neuraminidase and fusogenic activities of NDV. The balance among the three functions of HN determines the biological characteristics of NDV. We have also shown that the length of the HN protein cannot be reduced indefinitely and does not affect the virulence of NDV. These findings may also be applicable to other paramyxoviruses.

## Materials and Methods

### Viruses and cells

The recombinant viruses were generated in our laboratory from a full-length cDNA copy of the velogenic NDV strain rSG10, as described previously^47^. The NDV strain SG10 (an epidemic virulent strain in China of genotype VII) and V4 (an avirulent strain of genotype I) were used as the control. BSR T7/5 cells stably expressing the T7 phage RNA polymerase, a chicken embryo fibroblast cell line (DF-1), and an African green monkey kidney cell line (Vero) were all grown in Dulbecco’s modified eagle medium (DMEM, Gibco, Grand Island, NY, USA) with 10% fetal bovine serum (FBS, Gibco), and maintained in DMEM with 2% FBS at 37 °C under a 5% CO_2_ atmosphere.

### Mutation of full-length NDV cDNA and virus rescue

The previously generated full-length cDNA clone, pOK-rSG10-HN571^47^, was used as the backbone upon which to construct a series of mutants containing truncated or extended HN genes, as illustrated in Fig. 1A. To change the NDV HN protein length, the position of the stop codon in the HN ORF was altered with site-directed mutagenesis. All the used primer sequences are listed in Supplementary Table S2. The resulting full-length plasmids encoding the truncated or extended HN proteins were used to recover the recombinant viruses with a reverse genetics procedure. Virus rescue was performed as described previously^47^.

### Construction of HN and F protein expression plasmids

The HN and F genes of rNDV-SG10-HN571, rNDV-SG10-HN567, rNDV-SG10-HN577, rNDV-SG10-HN582 and rNDV-SG10-HN616 were amplified from full-length cDNA clones as described above by RT-PCR and were inserted into protein-expression plasmid pCI-neo (pCI) (Promega, Madison, USA) between the *XhoI* and *MluI* restriction sites. The plasmids obtained were named pCI-HN571, pCI-HN567, pCI-HN577, pCI-HN582, pCI-HN616 and pCI-F. Primers used to amplify the HN and F genes are listed in Supplementary Table S3.

### Reverse transcription (RT)-PCR, sequence analysis

Viral RNA was extracted from 100 μl of allantoic fluid with the Simply P Total RNA Extraction kit (BioFlux, Hangzhou, China), according to the manufacturer’s instructions. The fragments that included the altered HN sequences were amplified with RT-PCR using specific primers T-F and T-R (see Supplementary Table S2). The PCR products were sequenced directly with specific primers by Tsingke (Beijing, China). The nucleotide sequences were assembled, aligned, and compared with the DNASTAR program (Madison, WI, USA).

### Western blot analysis

Total cell protein lysates were extracted from infected or transfected Vero cells with ice-cold RIPA lysis buffer (50 mMTris [pH 7.4], 150 mM NaCl, 1% Triton X-100, 1% sodium deoxycholate). Cellular proteins were separated by 10% sodium dodecyl sulfate-polyacrylamide gel electrophoresis (SDS-PAGE) and transferred to a polyvinylidenedifluoride (PVDF) membrane (Amersham Biosciences, Germany). Each PVDF membrane was blocked with 5% (w/v) nonfat dry milk and 0.1% Tween 20 in Tris buffered saline (TBST) and subsequently incubated with a primary antibody overnight at 4 °C. Primary antibodies were SG10 polyclonal serum or anti-NDV F rabbit polyclonal antiserum (both diluted 1:100 and preserved in our laboratory), β-actin mouse monoclonal antibody (diluted 1:1000, Beyotime Biotechnology, Beijing, China). After being washed with TBST, the membranes were incubated for 1 h with corresponding horseradish peroxidase (HRP)-conjugated anti-chicken, anti-rabbit or anti-mouse antibody at a 1:10000 dilution (Bioss Biotechnology, Beijing, China). HRP presence was detected using a Western Lightning chemiluminescence kit (CWBIO, Beijing, China) in accordance with the manufacturer’s protocol. The load protein was normalized to β-actin. Protein bands were quantified by densitometry using ImageJ software (National Institute of Mental Health, Bethesda, MD, USA).

### Viral pathogenicity

The pathogenicity of the wild-type and recombinant viruses was determined with standard pathogenicity tests for NDV: MDT in 9-day-old embryonated SPF chicken eggs and ICPI in 1-day-old SPF chicks^2^. Their pathogenicity of the mutant NDVs in BSR T7/5 cells was examined by evaluating CPEs they exerted.

### Viral growth characteristics

The growth dynamics of nine NDVs, including the wild-type, parental, and recombinant viruses, were evaluated in DF-1 cells. The growth characteristics of five of these viruses were then confirmed in Vero cells. Cells in duplicate wells of six-well plates were infected with NDVs at an MOI of 0.01 PFU/cell, and then incubated with DMEM containing 2% FBS at 37 °C in 5% CO_2_. The culture supernatants were collected at 12 h intervals until 72 h after infection. The viral titers in the collected supernatants were quantified in DF-1 and Vero cells and expressed as median tissue culture infective doses (TCID_50_), using the endpoint method of Reed and Muench^56^.

### Hemagglutination (HA) titers of recombinant viruses and Hemadsorption (HAd) assay

HA titres of recombinant viruses at the different virus titers level (6.0 and 7.0 log_10_ TCID_50_/ml) were obtained by using the microplate HA test. The HAd activity was assayed as the ability of the recombinant viruses to adsorb to chicken red blood cells (CRBCs), as described previously^36^, with modifications. Confluent monolayers of Vero cells in 24-well plates were inoculated with virus at an MOI of 0.1 PFU/cell. At 24 h post-infection (hpi), the medium was removed and the cells were washed with cold PBS and then incubated for 30 min at 4 °C with a 2% (vol/vol) suspension of CRBCs. After the unbound CRBCs were removed with two washes of ice-cold PBS, the CRBCs bound to the virus-infected cells were lysed with a red-blood-cell lysis solution (0.145 M NH_4_Cl, 17 mM Tris-HCl). The lysate was clarified by centrifugation and transferred to 96-well plates. Absorbance was measured at 549 nm with a Spectramax M5 ELISA reader (Molecular Devices, Sunnyvale, CA, USA). The HAd assay at the protein level was performed with monolayers of Vero cells transfected using 0.5 μg each of pCI-HN mutant plasmids (pCI-HN571, pCI-HN567, pCI-HN577, pCI-HN582, pCI-HN616) as above described.

### NA assay

The NA activity was determined with a fluorimetric procedure, as described by Potier *et al*.^37^, with some modifications. Vero cell monolayers in 96-well plates were inoculated with NDVs at an MOI of 0.1 PFU/cell. At 24 hpi, the cells were washed with cold PBS and overlaid with 30 μl of substrate mix (one volume of 325 mM2-*N*-morpholinoethanesulfonic acid [MES; pH 6.4], two volumes of 0.5 mM 2′-(4-methylumbelliferyl)-α-D-N-acetylneuraminic acid [MUN; Sigma, St. Louis, MO, USA], and three volumes of 10 mM calcium chloride) per well, to give a final concentration of 100 μM MUN in the assay. Cells were incubated at 37 °C for 30 min with shaking, and the reaction was terminated by the addition of 0.014 M sodium hydroxide in 83% (vol/vol) ethanol. The fluorescence intensity was measured at an excitation wavelength of 360 nm and an emission wavelength of 450 nm with a Spectramax M5 ELISA reader (Molecular Devices). The NA assay at the protein level was performed with monolayers of Vero cells transfected using the same expression plasmids utilized in the HAd assay.

### Fusion index assay

The fusogenic abilities of the parental and recombinant viruses were examined in Vero cells, as described previously^22^. Confluent (10^6^ cells/ml) Vero cell monolayers in six-well plates were infected with NDVs at an MOI of 0.1 PFU/cell and maintained in DMEM with 2% FBS at 37 °C under 5% CO_2_. After the CPE was monitored for 48–72 h, the medium was removed and the cells were washed once with 0.02% EDTA, and then incubated with 1 ml of EDTA for 2 min at room temperature. The cells were washed with PBS and fixed with methanol for 20 min at room temperature, and then stained with hematoxylin-eosin. Fusion was quantitated with the fusion index: the ratio of the total number of nuclei to the number of cells in which these nuclei were observed (i.e., the mean number of nuclei per cell). The fusion index assay at the protein level was performed with monolayers of Vero cells co-transfected using 1 μg each of pCI-F and pCI-HN mutant plasmids. The HAd, NA and fusion index values for all the viruses or HN protein mutants were expressed as percentages of the values for the parental virus (rNDV-SG10-HN571) or the expressed HN571 protein (pCI-HN571), which were considered to be 100%.

### Hemolytic titrations

The hemolytic activities of the parental and recombinant viruses were performed according to previously published methods^57^. In brief, the allantoic fluids of the rescued viruses were clarified by centrifugation (4 °C, 20 min, 500 g). Supernatant fluids of different NDVs were diluted to the same hemagglutination unit/ml (HAU/ml) based on the HA titer. The diluted virus sample 0.5 ml was mixed with 1 ml of 1% suspension of chicken erythrocytes. After incubated on ice for 20 min with occasional shaking, the erythrocytes were sedimented by centrifugation at 500 g for 3 min, washed with phosphate-buffered saline (PBS) and resuspended in the same buffer (0.5 ml). After incubation for 60 min at 37 °C, tubes were then centrifuged at 200 g for 5 min. Supernatant fluids were then transferred to 96-well plates and their hemoglobin contents were measured as absorbance at 549 nm with a Spectramax M5 ELISA reader (Molecular Devices). As for the positive control, 0.03 M NH_4_OH was added to the assay and for the negative control, the PBS was used. The hemolytic values for all the viruses were expressed as percentages of the values for rNDV-SG10-HN571 at 2^8^ HAU/ml, which were considered to be 100%.

### Statistical analysis

All statistical significance of differences was performed using Prism 5.0 program (GraphPad Software Inc., San Diego, CA, USA). Statistical differences between repeats were determined using the analysis of variance (ANOVA) method followed by Tukey’s test. Statistical significance was set at p < 0.05 (*), p < 0.01 (**) and p < 0.001 (***), respectively.

## Additional Information

**How to cite this article**: Jin, J. *et al*. Contribution of HN protein length diversity to Newcastle disease virus virulence, replication and biological activities. *Sci. Rep.* **6**, 36890; doi: 10.1038/srep36890 (2016).

**Publisher’s note:** Springer Nature remains neutral with regard to jurisdictional claims in published maps and institutional affiliations.

## Supplementary Material

Supplementary Information

## Figures and Tables

**Table 1:** Pathogenicity of the parental and recombinant viruses.

Virus	MDT (h)^a^	ICPI score^b^
NDV-SG10-HN571	42.0	1.95
rNDV-SG10-HN571	42.0	1.93
rNDV-SG10-HN567	40.8	1.94
rNDV-SG10-HN568	42.0	1.93
rNDV-SG10-HN569	43.5	1.94
rNDV-SG10-HN570	39.0	1.95
rNDV-SG10-HN577	40.8	1.91
rNDV-SG10-HN582	40.0	1.99
rNDV-SG10-HN616	43.2	2.00

^a^MDT, the mean death times (hour) in embryonated chicken eggs. Criteria for classifying the virulence of NDVs: virulent viruses, <60 h; moderately virulent viruses, 60–90 h; avirulent viruses, >90 h.

^b^ICPI, intracerebral pathogenicity index, determined by inoculating groups of 10 1-day-old SPF chicks with fresh infective allantoic fluid *via* the intracerebral route. The birds were observed for clinical symptoms and mortality daily for 8 days. At each observation, the birds were scored 0 if normal, 1 if sick, and 2 if dead. The ICPI is the mean score per bird per observation. Criteria for classifying the virulence of NDVs: virulent viruses, 1.5–2.0; moderately virulent viruses, 0.7–1.5; avirulent viruses, 0.0–0.7.

**Table 2:** Hemagglutination (HA) titers of the parental and recombinant viruses.

Virus	HA titers^a^ (log_2_) of different virus titers^b^ (log_10_ TCID_50_/ml)	
	7.0	6.0
rNDV-SG10-HN571	5.17 ± 0.58	2.17 ± 0.58
rNDV-SG10-HN567	4.67 ± 0.29	1.17 ± 0.76
rNDV-SG10-HN577	5.33 ± 0.29	2.33 ± 0.29
rNDV-SG10-HN582	7.17 ± 0.58^*^	4.33 ± 0.29^*^
rNDV-SG10-HN616	6.17 ± 0.76	3.17 ± 0.58

^a^HA titers in microplate format were expressed in log_2_ of the mean ± standard deviation from three independent tests.

^b^Virus titers were expressed in log_10_ TCID_50_/ml on DF-1 cells. Asterisks at the same virus titer level indicate statistically significant differences (p < 0.05) of the HA titers compared with rNDV-SG10-HN571. P values were calculated with Tukey’s test (95% confidence levels).
