# Supplementary material for: Contribution of HN protein length diversity to Newcastle disease virus virulence, replication and biological activities
Source: Sci Rep. 2016 Nov 11;6:36890. doi: 10.1038/srep36890 (PMC5105081; doi:10.1038/srep36890)
Supplement: Supplementary Information [file srep36890-s1.doc]

**Contribution of HN protein length diversity to Newcastle disease virus virulence, replication and** [**biological**](../../../../../Owner/AppData/jjh/AppData/Local/Yodao/DeskDict/frame/20141206201354/javascript:void(0)%3B) [**activities**](../../../../../Owner/AppData/jjh/AppData/Local/Yodao/DeskDict/frame/20141206201354/javascript:void(0)%3B)

Jihui Jin, Jing Zhao, Yingchao Ren, Qi Zhong & Guozhong Zhang^*^

*Key Laboratory of Animal Epidemiology and Zoonoses, Ministry of Agriculture, College of Veterinary Medicine, China Agricultural University, Beijing 100193, People’s Republic of China*

^*^Corresponding author: College of Veterinary Medicine, China Agricultural University, No. 2 Yuanmingyuan West Road, Haidian District, Beijing 100193, People’s Republic of China. Tel.: +86-10-62733660; Fax: +86-10-62732984; E-mail: zhanggz@cau.edu.cn.


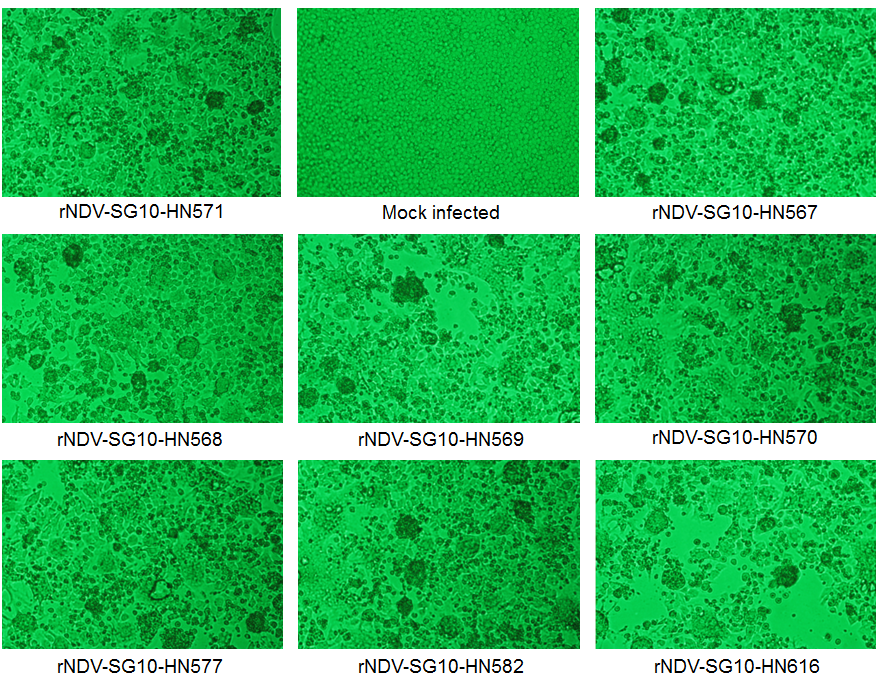
**Supplementary Fig. S1. CPEs of NDV strains in BSR T7/5 cells. BSR T7/5 monolayers were infected with NDV strains at an MOI of 0.01 PFU/cell. After 48 h, the CPE in each virus-infected monolayer was photographed under an inverted microscope.**

| **Group** | **No. of isolates** | **No. (%) of isolates with HN protein length** | | | | | | | | | | | |
| --- | --- | --- | --- | --- | --- | --- | --- | --- | --- | --- | --- | --- | --- |
|  |  | **570aa** | **571aa** | **572aa** | **577aa** | **578aa** | **580aa** | **581aa** | **582aa** | **585aa** | **586aa** | **615aa** | **616aa** |
| **Class І** | 109 | 0 | 0 | 3 (2.6)^b^ | 0 | 0 | 0 | 2 (1.8) | 0 | 21 (19.3) | 0 | 0 | 83 (76.1) |
| **Class II** |  |  |  |  |  |  |  |  |  |  |  |  |  |
| І | 69 | 0 | 0 | 0 | 2 (2.9) | 2 (2.9) | 10 (14.5) | 0 | 1 (1.4) | 0 | 1 (1.4) | 1 (1.4) | 52 (75.4) |
| II | 114 | 0 | 0 | 3 (2.6) | 102 (89.5) | 0 | 0 | 0 | 0 | 0 | 0 | 0 | 9 (7.9) |
| III | 9 | 0 | 9 (100) | 0 | 0 | 0 | 0 | 0 | 0 | 0 | 0 | 0 | 0 |
| IV | 14 | 0 | 14 (100) | 0 | 0 | 0 | 0 | 0 | 0 | 0 | 0 | 0 | 0 |
| V | 58 | 0 | 58 (100) | 0 | 0 | 0 | 0 | 0 | 0 | 0 | 0 | 0 | 0 |
| VI | 141 | 0 | 138 (97.9) | 0 | 3 (2.1) | 0 | 0 | 0 | 0 | 0 | 0 | 0 | 0 |
| VII | 422 | 2 (0.5) | 408 (92.3) | 6 (1.4) | 2 (0.5) | 0 | 0 | 0 | 4 (0.9) | 0 | 0 | 0 | 0 |
| VIII | 4 | 0 | 2 (50.0) | 0 | 0 | 0 | 0 | 1 (25.0) | 1 (25.0) | 0 | 0 | 0 | 0 |
| IX | 26 | 1 (3.8) | 25 (96.2) | 0 | 0 | 0 | 0 | 0 | 0 | 0 | 0 | 0 | 0 |
| **Total** | 966 | 3 (0.3) | 654 (67.7) | 12 (1.2) | 109 (11.3) | 2 (0.2) | 10 (1.0) | 3 (0.3) | 6 (0.6) | 21 (2.2) | 1 (0.1) | 1 (0.1) | 144 (14.9) |
| **Virulence**^c^ |  |  |  |  |  |  |  |  |  |  |  |  |  |
| Velogenic | 250 | 0 | 212 (84.8) | 11 (4.4) | 15 (6.0) | 0 | 7 (2.8) | 0 | 5 (2.0) | 0 | 0 | 0 | 0 |
| Lentogenic | 97 | 0 | 0 | 0 | 32 (33.0) | 2 (2.1) | 3 (3.1) | 0 | 1 (1.0) | 4 (4.1) | 0 | 0 | 55 (56.7) |
| **Total** | 347 | 0 | 212 (61.1) | 11 (3.2) | 47 (13.5) | 2 (0.6) | 10 (2.9) | 0 | 6 (1.7) | 4 (1.2) | 0 | 0 | 55 (15.9) |

**Supplementary Table S1. Summary of Newcastle disease virus strains possessing the corresponding HN protein length^a^.**

^a^ Sequence data were obtained from the National Center of Biotechnology Information (NCBI) (<http://www.ncbi.nlm.nih.gov/nuccore/>).

^b^ Number of isolates possessing a specific HN protein length (proportion of isolates with a specific HN protein length in the NDV of the corresponding NDV clades and virulence in the NCBI database).

^c^ Virulence was determined by the cleavage site amino acid sequence of the fusion protein.

**Supplementary Table S2. Primers for site-directed mutagenesis and sequencing.**

| **Primer** | **Location^a^** | **Nucleotide sequence^b^ (5’→3’)** |
| --- | --- | --- |
| P-F | 7978-8000 | AGCACCAAGGCAGCATACACAAC |
| P-R | 11495-11516 | CTTCCATGATAGCATGTGCGAC |
| 565-F | 8087-8134 | GGATCGTTCCCTTATTAGTTGAGATC**TAG**AAGGATGATAGAGTTTAAG |
| 565-R | 8087-8128 | CTCTATCATCCTT**CTA**GATCTCAACTAATAAGGGAACGATCC |
| 566-F | 8104-8134 | GTTGAGATCCTC**T**AGGATGATAGAGTTTAAG |
| 566-R | 8096-8128 | CTCTATCATCCT**A**GAGGATCTCAACTAATAAGG |
| 567-F | 8104-8138 | GTTGAGATCCTCAAG**T**A**G**GATAGAGTTTAAGAAGC |
| 567-R | 8096-8128 | CTCTATC**C**T**A**CTTGAGGATCTCAACTAATAAGG |
| 568-F | 8107-8144 | GAGATCCTCAAGGAT**T**A**G**AGAGTTTAAGAAGCTAGACT |
| 568-R | 8104-8141 | CTAGCTTCTTAAACTCT**C**T**A**ATCCTTGAGGATCTCAAC |
| 569-F | 8106-8147 | TGAGATCCTCAAGGATGAT**T**GAGTTTAAGAAGCTAGACTTGG |
| 569-R | 8104-8144 | AGTCTAGCTTCTTAAACTC**A**ATCATCCTTGAGGATCTCAAC |
| 570-F | 8112-8150 | CCTCAAGGATGATAGA**TAA**TAAGAAGCTAGACTTGGCCG |
| 570-R | 8108-8146 | CAAGTCTAGCTTCTTA**TTA**TCTATCATCCTTGAGGATCT |
| 577-F | 8125-8164 | AGAGTTT**T**AGAAGCTAGACTTGGC**T**GATTGAGCCAATCAT |
| 577-R | 8128-8168 | TCCTATGATTGGCTCAATC**A**GCCAAGTCTAGCTTCT**A**AAAC |
| 582-F | 8113-8151 | CTCAAGGATGATAGAGTTT**T**AGAAGCTAGACTTGGCCGA |
| 582-R | 8112-8150 | CGGCCAAGTCTAGCTTCT**A**AAACTCTATCATCCTTGAGG |
| 616-F1 | 8248-8286 | CATGTTGTTGGGCAGCCA**T**AATCAGACAATGCTGATATG |
| 616-R1 | 8143-8183 | TCGTCTTCCCAACCATCC**A**ATGATTGGCTCAATCGGCCAAG |
| 616-F2 | 8205-8267 | CAATGCTT**T**GAGTCAAGCTGAATATT**T**ACATAAGCCAGGATCCCATGTTGTTGGGCAGCCA**T**A |
| 616-R2 | 8163-8224 | CAGCTTGACTC**A**AAGCATTGTGGGAGATGATTGGTGCGGTGTCGTCTTCCCAACCATCC**A**AT |
| T-F | 7787-7807 | CATCAGCAAGATGCCCCAACT |
| T-R | 9057-9077 | GGGTGAGGCATGTGAACTTGT |

^a^ Primer locations are listed according to NDV strain SG10.

^b^ Altered nucleotides are indicated in bold.

**Supplementary Table S3. Primers for construction of HN and F protein expression plasmids.**

| **Primer** | **Nucleotide sequence^a^ (5’→3’)** |
| --- | --- |
| pCI-HN-F | CCG***CTCGAG***ATGGACCGCGCGGTTAACAGAGTC |
| pCI-HN571-R | CG***ACGCGT***TTAAACTCTATCATCCTTGAGGATCT |
| pCI-HN567-R | CG***ACGCGT***CTACTTGAGGATCTCAACTAATAAGGGAACG |
| pCI-HN577-R | CG***ACGCGT***TCAGCCAAGTCTAGCTTCTAAAAC |
| pCI-HN582-R | CG***ACGCGT***CTATGATTGGCTCAATCGGCCAAG |
| pCI-HN616-R | CG***ACGCGT***TTATGGCTGCCCAACAACATGGGATC |
| pCI-F-F | CCG***CTCGAG***ATGGGCTCCAAACCTTCTACCAGGAT |
| pCI-F-R | GC***ACGCGT***TCATGCTCTTGTAGTGGCTCTCATCT |

^a^ *Xho*I and *Mlu*I restriction sites are indicated in bold italics.
